# Update on the Biology, Management, and Treatment of Small Cell Lung Cancer (SCLC)

**DOI:** 10.3389/fonc.2020.01074

**Published:** 2020-07-16

**Authors:** Andreas Saltos, Michael Shafique, Alberto Chiappori

**Affiliations:** Department of Thoracic Oncology, H. Lee Moffitt Cancer Center and Research Institute, Tampa, FL, United States

**Keywords:** small cell lung cancer, immunotherapy, chemotherapy, checkpoint inhibitors, vaccines

## Abstract

Small-cell lung cancer (SCLC) accounts for 13–15% of all new lung cancer cases in the US. The tumor has a tendency to disseminate early resulting in 80–85% of patients being diagnosed with extensive disease (ES-SCLC). Chemotherapy has provided SCLC patients considerable survival benefits over the past three decades. Nonetheless, most patients relapse and rarely survive beyond 2 years. Despite consistent overall response rates of ≥50%, until recently, median survival times and 2-year survivals only ranged between 7–10 months and 10–20%, respectively. Several chemotherapy agents possess activity against SCLC, both, as single agents and in combinations but etoposide-platinum emerged as the preferred first line regimen. Upon relapse, many patients remain candidates for additional therapy. However, the sensitivity of relapsed SCLC to further therapies is markedly reduced and dependent upon the level and duration of response to the initial treatment (platinum-sensitive vs. resistant relapse). Multiple factors suggest a therapeutic role for immunotherapy in SCLC:

1. SCLC has been associated with immune-mediated paraneoplastic processes (cerebellar degeneration, limbic encephalitis, and Lambert–Eaton syndrome) and patients presenting with these paraneoplastic syndromes have shown more favorable outcomes, suggesting an underlying immune response mechanism.
2. Comprehensive genomic profiling of SCLC indicates that the majority lack functional p53 (90%) and Rb1 (65%). These universal genetic aberrations facilitate poor genomic stability, thus perpetuating the generation of tumor associated antigens, amenable to targeting with immunotherapy.
3. SCLC has one of the highest mutational loads, likely a reflection of the myriad of insults inflicted by smoking-related carcinogens. The relationship between tumor mutational load and response to immune checkpoint inhibitors has been established in multiple solid tumors, including preliminary results in relapsed SCLC.

In this manuscript, we review the early (some failed and discontinued, some partly successful, and still ongoing) attempts to incorporate immunotherapy (particularly vaccine based approaches) to the treatment of SCLC, and the latest attempts (mostly incorporating the use of checkpoint inhibitors), including those with favorable but preliminary results (CheckMate 032, Keynote 028 and 158), and those with more definitive positive (iMpower 133 and CASPIAN) and negative (CheckMate 331 and 451) results.

## Introduction

In 2020, an estimated 228,820 new cases of lung cancer (LC) will be diagnosed in the United States (US) and ~135,720 patients with LC will die from the disease (1). Small-cell LC (SCLC) accounts for 13–15% of all new LC cases (2) and represents the sixth most commonly diagnosed cancer in the US. The tumor has a tendency to disseminate early, so that 80–85% of patients present with advanced or extensive disease (ES-SCLC) at diagnosis (3). Given the systemic nature and recognized (initial) chemo-sensitivity of SCLC, considerable survival improvements are obtained with platinum-based chemotherapy; the cornerstone of treatment management for SCLC patients for more than 3 decades (3). Nonetheless, most ES-SCLC patients relapse and rarely survive beyond 2 years (10–20%), and despite overall response rates (ORR) of ≥50%, median survival time (4) only ranges from 7 to 10 months (5).

In the US, etoposide-platinum (EP) is the preferred first-line regimen and attempts to improve outcomes utilizing multiple different strategies have consistently failed (3, 5). Many relapsed patients remain candidates for additional therapy. However, their benefit from further therapies is markedly reduced (MST varies from 25 to 32 weeks) and dependent on the response to initial treatment (platinum sensitive vs. resistant relapse) (6).

SCLC has classically been associated with immune-mediated paraneoplastic processes, such as cerebellar degeneration, limbic encephalitis, and Lambert-Eaton syndrome (7). For example, antibodies generated against human neuronal RNA-binding proteins (e.g., Hu), expressed on neurons and SCLC, lead to encephalomyelitis (8). Interestingly, SCLC patients that present with these “early” paraneoplastic syndromes have a more favorable prognosis (9, 10), suggesting that an underlying immune response is being generated against these onconeural antigens.

Comprehensive genomic profiling has identified lack of functional p53 and Rb1 (11, 12) and amplification of MYC in the vast majority of SCLC tumors. These two defective tumor suppressor genes (p53 and Rb1), along with aberrant expression of the MYC oncogene, lead to rapid proliferation and, consequently, to replication stress (13, 14). These genetic aberrations facilitate poor genomic stability (15) and perpetuate the generation of tumor-associated antigens (TAAs).

Furthermore, among solid tumors, SCLC is also known to have one of the highest tumor mutational burdens (TMB) (16), which is thought to be a reflection of the myriad of insults inflicted by smoking-related carcinogens but, more importantly, potentially predicts sensitivity to checkpoint inhibitors. This predictive potential was first shown in metastatic melanoma (17) and NSCLC (18, 19) but it was also suggested in SCLC where a survival analysis by TMB from CheckMate 032 showed a benefit for those patients in the highest TMB tercile (20).

In this manuscript, we review the early (some failed and discontinued, some partly successful, and still ongoing) attempts to incorporate immunotherapy (particularly vaccine based approaches) to the treatment of SCLC and the latest attempts (mostly incorporating the use of checkpoint inhibitors), including those with favorable but preliminary results (CheckMate 032, Keynotes 028 and 158) and those with more definitive positive (iMpower 133 and CASPIAN) and negative (CheckMate 331 and 451) results.

## Immunotherapy Approaches Prior to Checkpoint Inhibition

For many years prior to the era of immune checkpoint inhibitors (ICI), very little progress was made in the treatment of SCLC despite trials of many emerging therapeutic approaches. Among these, a number of immunomodulatory agents have been subjects of investigation in SCLC, sparked by the observation of antitumor activity with immunotherapies in other cancers. These strategies included administration of recombinant cytokines, other immunomodulatory agents, as well as anti-tumor vaccines. Although early studies failed to translate into any approved therapies, a number of trials hinted at the potential of this approach in SCLC.

### Immunomodulatory Agents

Interleukin-2 (IL-2) is a cytokine which plays a key role in T-cell growth and differentiation. Building on the observation that high dose recombinant IL-2 could induce immune responses and durable remissions in a subset of patients with metastatic melanoma or renal cell carcinoma (21), trials using this agent in SCLC were also undertaken. The CALGB conducted a phase II trial of IL-2 in patients with ES-SCLC who had persistent measurable disease after induction chemotherapy with at least one cycle of PACE (cisplatin, doxorubicin, cyclophosphamide, and etoposide) (22). Patients were treated with IL-2 as a continuous infusion at ~12 million IU/m^2^/day for 96 h, followed by a 3-day rest, with planned 8-week duration of therapy. Interestingly, 4/24 (17%) of these patients went on to attain a complete response after IL-2 therapy. However, only 5/24 (21%) patients were able to complete the planned 8 weeks of therapy, primarily due to toxicity (22). A subsequent phase II trial tested IL-2 in the pre-chemotherapy setting (23). Ten patients with newly diagnosed ES-SCLC were given two cycles of subcutaneous IL-2 at 12 million IU/m^2^ daily on days 1–5 and 8–12 every 4 weeks. The trial was terminated early due to a lack of efficacy, with only one partial response among the first 10 patients and death of one patient due to rapid progression that precluded chemotherapy. Ultimately, due to the high rate of toxicity and poor response rate, this therapy was not pursued further.

Interferons (IFNs) can exhibit anticancer effects through a variety of mechanisms, including stimulation of immune response as well as direct antiproliferative effects through inhibition of angiogenesis and endothelial growth factors (24, 25). A phase III trial published in 1992 by Mattson et al. was among the first to investigate low dose IFN-α as maintenance therapy in SCLC. All patients on this trial had first completed chemotherapy (four cycles of cyclophosphamide, vincristine, and etoposide) followed by split-course RT (55 Gy in 20 fractions over 7 weeks) (26). Patients were then randomized between three (3) arms receiving either maintenance low-dose IFN-α, maintenance chemotherapy (six cycles of cyclophosphamide, doxorubicin, and cisplatin), or no maintenance therapy. While no difference in median survival was noted in any of the arms (11 vs. 11 vs. 10 months), a subset analysis suggested that long-term survival was improved for those patients with LS SCLC (26). However, a series of subsequent trials of recombinant IFN-α or IFN-γ as maintenance therapy in SCLC, in both the limited and extensive stage settings, failed to demonstrate any improvement in progression-free or overall survival (27–30).

The combination of IFN plus chemotherapy has also been tested in multiple trials with mixed results. In a randomized trial conducted in the early 1990s, 90 SCLC patients were assigned to receive chemotherapy with or without combination IFN-α2a (given 3 MU/m^2^ twice weekly) (31). The arm receiving chemotherapy plus IFN experienced a higher rate of complete response (38 vs. 28%), as well as a statistically significant improvement in overall survival among patients with limited-stage disease (*p* < 0.0067) (31). A randomized phase III trial compared cisplatin and etoposide chemotherapy with or without IFN-α (3 MU/m^2^ three times weekly IM), and demonstrated no difference in median survival, with a trend for inferior 2-year survival rates in those treated with IFN, along with higher rates of dose-reduction of chemotherapy in the IFN group due to myelosuppression (32). Additional trials investigated the combination of IFN-α with 13-cis-retinoic acid concurrent with chemotherapy, without statistically significant improvements in survival (30, 33). A more recent phase II study randomized 164 patients with untreated SCLC to four possible treatment arms: chemotherapy alone (carboplatin, ifosphamide, and etoposide) or combined with IFN-α, IFN-γ, or IFN-α plus IFN-γ (34). No significant differences in response rates or OS were seen between the treatment arms, although a subset analysis of only limited stage patients in the IFN-α arm suggested a possible benefit. Patients treated with the IFN combinations experienced higher rates of fever, anorexia, and fatigue (34).

Toll-like receptor 9 (TLR9) is expressed on a variety of immune cells and plays a major role in activation of innate immunity including stimulation of cytokine production including type 1 IFNs (35). Lefitolimod (MGN1703) is a DNA molecule which functions as a TLR9-agonist and demonstrated favorable tolerability and evidence of anti-tumor immune activation in early studies (35, 36). The randomized, phase II IMPULSE trial tested lefitolimod as maintenance in ES-SCLC. Patients who had an objective response following four cycles of platinum-based first-line induction chemotherapy were randomized to local standard-of-care as maintenance vs. lefitolimod (60 mg subcutaneous twice weekly). Although there was no observed advantage in median overall or progression-free survival on the lefitolimod arm, there was a signal of benefit in prespecified subgroups including patients with a low frequency of activated CD86+ B cells (HR 0.59, 95% CI 0.29–1.21). Interestingly, two (2) patients in the lefitolimod arm remained progression-free at 2 years of follow-up. Treatment with lefitolimod was well tolerated, with the most common reported adverse effects being cough (25%), headache (23%), and fatigue (18%) (36).

### Tumor Vaccines in SCLC

Tumor vaccines have also been utilized as a distinct approach to stimulating antitumor immunity by allowing tumor antigen presentation to immune cells with the goal of generating an adaptive immune response. A number of vaccine approaches have been previously tested in clinical trials in SCLC. Although there have been no therapeutic approvals for this setting, some interesting results suggest a role for this strategy in SCLC.

GD3 is a cell-membrane ganglioside which is expressed in the majority of cases of SCLC and with limited expression in normal tissues. Vaccination with the anti-idiotypic monoclonal antibody BEC2 (which had been shown to induce anti-GD3 antibodies) plus Bacillus Calmette–Guerin (BCG) vaccine was investigated in SCLC after evidence of enhanced immunogenicity using this approach in melanoma (37). Fifteen (15) patients who had completed standard therapy with partial or complete response were given a series of five immunizations of BEC2 and BCG administered intradermally on weeks 0, 2, 4, 6, and 10. A considerably longer median relapse-free survival was observed among these patients compared to historical controls (11 months for ES-SCLC and >47 months for LS-SCLC) (38). While all 15 patients developed anti-BEC2 antibodies, five (5) patients developed detectable anti-GD3 antibodies which were associated with longer relapse-free survival. A randomized phase III trial of BEC2 and BCG administered after completion of chemoradiation therapy in 515 patients with LS-SCLC was reported in 2005 by Giaccone et al. (39). Unfortunately, there was no observable improvement in overall survival (OS), progression-free survival (PFS), or quality of life (QoL) for patients in the vaccination arm. One possible explanation was that only one third of patients developed a humoral response to the vaccine. Among that subset of patients, there was a trend toward prolonged survival (19.2 vs. 13.9 months, *P* = 0.085), although not statistically significant (39).

Another tumor vaccine utilizing the anti-idiotypic monoclonal antibody 1E10, which also seeks to illicit an immune response against the GM3 ganglioside, was developed for use in SCLC as well as melanoma and breast cancer (40). A phase I clinical trial of 1E10 in SCLC was conducted in nine (9) patients who had a response to their initial induction chemotherapy (41). 1E10 was administered as four (4) biweekly injections followed by another six (6) doses at 28-day intervals. These patients had a prolonged survival compared with historical controls (including two of three patients with ES-SCLC with survival beyond 20 months and three of six with LS-SCLC beyond 40 months). A measurable immune response against GM3 was detected in seven out of eight of the patients evaluable for immunogenicity. The vaccine was well-tolerated with toxicities primarily consisting of grade 1–2 injection site reactions, with no grade 3 events (41). However, further development of 1E10 (now called racotumomab) was directed in NSCLC rather than SCLC (42), and has not been pursued further.

N-polysialic acid (polySA) is a polymer side chain bound to the neural cell adhesion molecule that is commonly expressed in SCLC (43). A phase I study of N-(polySA) conjugated to keyhole limpet hemocyanin (KLH) vaccine in SCLC induced a robust antibody response. However, peripheral neuropathy and ataxia were limiting toxicities at a dose of 30 μg, and a subsequent de-escalation study established 10 μg of N-(polySA) as the lowest optimally immunogenic dose (44). Post-vaccination sera from six of nine patients treated at this dose reacted strongly with human SCLC cells. Self-limited grade 3 ataxia of unclear etiology was seen in 1 of 18 patients. Subsequent development of this vaccine has also been limited.

Mutated p53 protein in SCLC induces MHC class I expression of p53-derived epitopes on the tumor cell surface, making it an attractive target for immune recognition (45, 46). In light of this, a tumor vaccine consisting of autologous dendritic cells transduced with wild-type p53 gene delivered by an adenovirus (Ad.p53 DC) was developed and tested in a Phase I/II trial in patients with ES-SCLC who had progressed on prior chemotherapy (47). Although a clinical partial response was observed in only one of the 29 patients, a p53-specific immune response was detected in ~57% of patients and, more interestingly, a high rate (62%) of objective clinical responses to subsequent (salvage) cytotoxic chemotherapy was noted (47).

A subsequent randomized phase II trial enrolled 69 patients with ES-SCLC who had completed 4–6 cycles of initial platinum plus etoposide chemotherapy to receive observation, the Ad.p53 DC vaccine, or vaccine plus all-trans-retinoic acid (ATRA) (48). ATRA was selected based upon evidence of its ability to suppress myeloid-derived suppressor cells. The vaccine was administered every 2 weeks for three doses, and all patients received paclitaxel upon progression. The primary endpoint was overall response rate (ORR) to paclitaxel. The ORR to subsequent chemotherapy was 15.4 vs. 16.7 vs. and 23.8%, in the observation, vaccine, and vaccine plus ATRA arms, respectively—although this difference was not statistically significant. In addition, positive anti-p53 immune responses were seen in 20% of patients treated with vaccine, and 43.3% of those treated with vaccine plus ATRA (48). Subsequently, due to the failed translation of therapeutic approaches to approved therapies despite hints of positive findings, the growth of checkpoint inhibition offered new opportunities for therapeutic advancement in SCLC.

## Current Clinical Practice: Immunotherapy Approaches and Checkpoint Inhibition

Clinicians have a wide-array of checkpoint inhibitors for use in extensive stage small cell lung cancer (ES-SCLC), both in the recurrent and the frontline setting. Additional approvals are likely. We review the clinical trial data supporting the use of these agents in ES-SCLC.

### Nivolumab

Nivolumab is a humanized IgG4 monoclonal antibody targeting the programmed death protein (PD-1), and many clinical trials have investigated the use of nivolumab in ES-SCLC (49). Checkmate-032 is a multi-center phase 1/2 trial which studied the safety and preliminary efficacy of nivolumab as monotherapy or in combination with ipilimumab in patients with recurrent small cell lung cancer (50). The Checkmate-032 trial was initially designed with a non-randomized cohort only and, subsequently, investigators added a randomized cohort in order to assess nivolumab monotherapy vs. the combination of ipilimumab and nivolumab.

Investigators reported the results of nivolumab monotherapy as a third-line or later treatment using pooled results of patients in both the randomized and non-randomized cohorts who received nivolumab monotherapy. It is important to note that the inclusion criteria for the SCLC cohorts of Checkmate-032 specified progression after one (1) or more platinum-based chemotherapy regimens, and that the nivolumab monotherapy results here include patients who received two (2) or more prior chemotherapy regimens (51). Patients received nivolumab 3 mg/kg every 2 weeks. In this heavily pre-treated population, numbering 109 patients, nivolumab monotherapy demonstrated an ORR of 11.9% with a median duration of response of 17.9 months. Based on this data, the FDA approved nivolumab for use in patients with ES-SCLC progressing after platinum-based chemotherapy and one other line of treatment.

Checkmate-331 reported the results of nivolumab as a second-line treatment for SCLC following recurrence after platinum-based chemotherapy (52). This trial is a randomized, open-label study of nivolumab monotherapy vs. topotecan in relapsed SCLC following platinum-based chemotherapy. A total of 569 patients participated with 284 receiving nivolumab. Overall survival was the primary endpoint. The trial did not meet this endpoint, demonstrating a hazard ratio of death of 0.86 (95% CI, 0.72–1.04). In the subgroup of patients with platinum-resistant disease, however, the HR was statistically significant (HR 0.71; 95% CI, 0.54–0.94). The authors also noted a delayed separation in the survival curves at 12-months. Despite some encouraging aspects to this trial, nivolumab is currently not approved as a second-line therapy.

### Ipilimumab

Ipilimumab is a human IgG1 monoclonal antibody that targets the CTLA-4 antigen, found on activated T cells (49). When not paired with nivolumab, ipilimumab has overall demonstrated limited activity in ES-SCLC. A phase II study first investigated the addition of ipilimumab to standard chemotherapy (53). Patients received, in a 1:1:1 randomized fashion, one of three regimens: (1) carboplatin, paclitaxel, (2) concurrent ipilimumab 10 mg/kg with carboplatin and paclitaxel, (3) phased ipilimumab with carboplatin and paclitaxel, where ipilimumab was started with cycle 3 of treatment. Primary endpoints included PFS, irPFS, and OS. This study enrolled 130 patients, and though irPFS was improved with phased ipilimumab when compared to the control arm, the other endpoints, including PFS and OS, showed no statistical difference. In a follow up phase III study, patients with ES-SCLC received either platinum and etoposide chemotherapy and placebo or platinum, etoposide, and ipilimumab 10 mg/kg in a phased regimen followed by maintenance ipilimumab (54). The study enrolled 1,132 patients, making it the largest study in ES-SCLC at the time (55). Nine hundred and fifty-four patients received treatment. Median OS was not statistically significant between the two groups. Patients in the control arm demonstrated a median OS of 10.9 months, compared to 11.0 months in the phased ipilimumab group, representing a hazard ratio of 0.94 (95% CI, 81–1.09; *p* = 0.3775). The authors reported an 18% treatment discontinuation rate among patients in the phased ipilimumab group. Similar numbers of patients dropped out, most commonly due to disease progression, within the first two cycles, prior to administration of placebo or ipilimumab (13% in the study arm, 17% in the control).

### Ipilimumab and Nivolumab

Our understanding of the clinical utility of combination ipilimumab and nivolumab has evolved over time, from when the safety and efficacy of this regimen was first reported. Checkmate-032 was an international, multi-center, two-stage multi-arm phase 1/2 study (50). Patients with progressive SCLC enrolled onto one of four different treatment arms: nivolumab monotherapy (3 mg/kg every 2 weeks) or one of the combination arms. The combination arms represented different dose levels, with dose level 1 consisting of nivolumab 1 mg/kg + ipilimumab 1 mg/kg (N1I1) every 3 weeks for four doses. If dose level 1 confirmed the safety of this combination, patients subsequently enrolled onto dose level 2, where they received nivolumab 1 mg/kg + ipilimumab 3 mg/kg (N1I3). Dose level 2b included nivolumab 3 mg/kg ipilimumab 1 mg/kg (N3I1). In all combination arms, patients received ipilimumab + nivolumab every 3 weeks for four cycles followed by maintenance nivolumab monotherapy 3 mg/kg every 2 weeks. The primary endpoint of this study was efficacy, assessed by the proportion of patients with objective responses. The study included all enrolled patients in the efficacy analysis.

At the time of publication in 2016, 98 patients had received nivolumab monotherapy. Three patients were treated on the N1I1 arm, as a preliminary demonstration of safety. Sixty-one patients and 54 patients received N1I3 and N3I1, respectively. Each of the three combination arms demonstrated the safety and tolerability of these regimens. However, grade 3 and 4 toxicities were highest in the N1I3 dose level, with 30% of patients experiencing such side effects. The ORR in the N monotherapy, N3I1, and N1I3 arms were 10% (95% CI, 5–18), 19% (95% CI, 9–31), and 23% (95% CI, 13–36), respectively. The median OS results in these three arms were 4.4 months (95% CI, 3.0–9.3), 7.7 months (95% CI, 3.6–18.0), and 6.0 months (95% CI, 3.6–11.0), respectively.

Following these results, Checkmate-032 included an expansion cohort for SCLC patients in which patients were randomized 3:2 to either nivolumab monotherapy vs. N1I3. A total of 243 patients enrolled in this expansion cohort, and 147 patients received nivolumab monotherapy (56). Ninety-six patients received the N1I3 combination. Initial results were promising at an ASCO 2017 presentation, with a non-statistical trend to improved OS. However, final analysis showed a median OS of 5.7 months (95% CI, 3.8–7.6) for the nivolumab monotherapy arm vs. 4.7 months (95% CI, 3.1–8.3) for the combination arm. Objective response rates favored N1I3, however, 21.9 vs. 11.6% (odds ratio 2.1; 95% CI, 1.06–4.26; *p* = 0.03).

Checkmate-451 assessed ipilimumab and nivolumab in the maintenance setting (57, 58). Patients could enroll if they received four (4) cycles of platinum-based chemotherapy. Patients received either N1I3, nivolumab monotherapy 240 mg every 2 weeks, or placebo upon randomization in a 1:1:1 fashion. The primary endpoint was OS of N1I3 vs. placebo. Eight hundred and thirty-four patients enrolled on this study, and the study did not meet its primary endpoint. The HR for overall survival was 0.92 (95% CI, 0.75–1.12; *P* = 0.3693). Notably, nearly one-third of patients in the N1I3 arm discontinued treatment due to toxicity, and seven (7) patients experienced treatment-related death.

### Atezolizumab

NCT01375842 investigated the use of atezolizumab in ES-SCLC in a small, Phase 1a study (59). Investigators enrolled and treated 17 patients with heavily pre-treated ES-SCLC. The dose of atezolizumab was either 15 mg/mg or 1,200 mg every 3 weeks, depending on the protocol amendment in use at the time. In addition, the trial initially selected for PD-L1 expression, but relaxed this inclusion criterion in subsequent amendments. Eight (47%) of patients had grade 3 or higher adverse events (AEs), including 1 episode of grade 5 hepatic failure. Generally, the safety profile was felt to be acceptable. This small trial did produce some encouraging efficacy signals. Though the ORR was just 6%, by RECIST criteria, the duration of treatment for some patients was encouraging. Two patients remained on study for ≥12 months. The median PFS and OS in this small study was in line with what has been previously reported with checkpoint inhibitor monotherapy.

Atezolizumab further demonstrated efficacy in the first-line setting in ES-SCLC (60). IMpower 133 is a phase 3, randomized, placebo-controlled trial which investigated the combination of atezolizumab with platinum-based chemotherapy (CT) with carboplatin and etoposide (A+CT) and compared outcomes to CT alone. The protocol randomized patients 1:1 to A+CT or CT alone for four, 21-day cycles of treatment followed by maintenance with A or placebo every 21 days until disease progression or unacceptable toxicity. Patients received standard doses of the investigational agents, including carboplatin AUC 5 mg/ml per min, etoposide 100 mg/m^2^, and atezolizumab 1,200 mg. IMpower133 defined two primary endpoints, investigator assessed OS and PFS in the intention-to-treat population.

Over 400 patients were ultimately randomized and treated, with 201 receiving A+CT and 202 receiving CT alone. The trial met both primary endpoints, demonstrating improvements in OS and PFS. Patients receiving A+CT demonstrated a median OS of 13.9 months compared to 10.3 months in the CT arm, representing a HR of death of 0.70 (95% CI, 0.54–0.91; *p* = 0.007). The HR for progression or death was 0.77 in favor of the A+CT arm (95% CI, 0.62–0.96; *p* = 0.02). Based on this data, the FDA approved atezolizumab for use in combination with carboplatin and etoposide in the first-line treatment of ES-SCLC.

Despite the improvement in survival seen with atezolizumab use, the response rates between the two regimens were not markedly different (60.2% with atezolizumab vs. 64.4% with placebo). AEs of Grade 3 or higher occurred in 58.1% of patients with atezolizumab vs. 57.6% of patients with placebo. AEs leading to discontinuation of any treatment were 11.1% with atezolizumab and 3.1% with placebo. Immune related AEs occurred in 39.9% of patients receiving atezolizumab compared to 24.5% of those receiving placebo. The rate of Grade 3–4 immune-related AEs was 10.6% in patients receiving atezolizumab and 2.6% in those receiving placebo.

Additionally, several important subgroups were analyzed, including patients with brain metastases (asymptomatic and treated prior to enrollment) and liver metastases. The median OS trended toward favoring atezolizumab in those patients with liver metastases (HR 0.81; 95% CI, 0.55–1.20). In those patient with treated brain metastases, however, the hazard ratio actually favored placebo (HR 1.07; 95% CI, 0.47–2.43). The small number of patients with brain metastases included (*n* = 9) may explain this result. Exploratory analysis assessing TMB as a predictive marker for OS was generally favorable. The HR for TMB ≥ 16 mutations/MB and ≥10 mutations/MB were 0.63 (95% CI, 0.35–1.15) and 0.68 (95% CI, 0.47–0.97), respectively.

### Durvalumab

Durvalumab is a human IgG1 monoclonal antibody targeting the programmed death protein ligand (PD-L1) (49). In NCT01693562, a small Phase 1/2 study, investigators enrolled patients with ES-SCLC to a regimen of durvalumab 10 mg/kg q2W (61). The patients had received a median of two lines of prior chemotherapy. No grade 3 or higher AEs were reported, and all AEs were either grade 1 or 2 in severity. This small study reported an ORR of 9.5%, with two (2) patients experiencing PR.

Following demonstration of safety, durvalumab demonstrated efficacy in the first-line setting. CASPIAN is a phase 3, randomized, open label clinical trial which demonstrated a survival advantage for the combination of durvalumab (D) with standard doses of carboplatin (or cisplatin) and etoposide compared to standard platinum-based chemotherapy alone (4). It contained three treatment arms, D+CT, D+tremelimumab (T)+CT, and CT alone. In 2019, the investigators reported the OS data in the D+CT arm compared to the CT alone arm. Patients received etoposide at doses of 80–100 mg/m^2^ on days 1–3 with either carboplatin AUC 5–6 mg/mL per min or cisplatin 75–80 mg/m^2^ on day 1. Patients in the D+CT arm received 1,500 mg of durvalumab on day 1. CASPIAN demonstrated a significant improvement in overall survival among the D+CT arm, with median OS of 13.0 months compared to 10.3 months in the CT along arm, representing a HR of death of 0.73 (95% CI, 0.50–0.91; *p* = 0.0047). Based upon this trial, the FDA approved the use of durvalumab (but not tremelimumab) in combination with etoposide and either carboplatin or cisplatin (62).

### Tremelimumab and Durvalumab

Tremelimumab is a human IgG2 monoclonal antibody targeting CTLA-4 (49). It has demonstrated safety when used in combination with durvalumab in multiple solid tumors, including ES-SCLC. In NCT02537418, investigators combined D±T with a variety of platinum-based chemotherapy doublets in patients with all solid malignancies (63). Platinum agents included carboplatin or cisplatin. Additional agents tested were pemetrexed, nab-paclitaxel, gemcitabine, and etoposide. Of the 118 patients in this safety analysis of irAEs, 78 had thoracic malignancies including NSCLC and SCLC. The safety analysis did not demonstrate any concerning safety signals.

Durvalumab and tremelimumab demonstrated acceptable safety and encouraging antitumor activity in patients with ES-SCLC with who were platinum resistant or refractory. Arm A of the BALTIC study reported data on 25 patients who received D 1500 q4W + T75 mg q4W for up to four cycles (16 weeks) followed by D 1500 mg q4W as maintenance or monotherapy (64). Ten patients experienced treatment related AEs of grade 3 or higher. Four patients (19%) experienced AEs grade 3 or higher attributable to the study treatments. Only one patient discontinued D+T due to a treatment related AE. The combination demonstrated an ORR of 9.8%, both of which were confirmed partial responses. An additional patient experienced an unconfirmed partial response. Five patients experienced SD as their best response.

NCT02261220 studied an alternative dosing regimen to flat dose D+T (65). In this study, investigators enrolled patients with ES-SCLC to receive D 20 mg/kg + T 1 mg/kg q4W for seven cycles (28 weeks) followed by D+T and the same doses q12W for two more doses, finally followed by maintenance or monotherapy D 10 mg/kg. This trial reported on 30 patients who received this regimen, all of whom had prior platinum-based chemotherapy for SCLC and 19 (63%) of whom were platinum resistant or refractory. Seven (23%) of patients experienced grade 3 or 4 treatment related AEs, and no patients discontinued D+T due to treatment related AEs. This regimen demonstrated an ORR of 13.3%, with two patients obtaining a CR.

As discussed in section Durvalumab, the CASPIAN study contained a durvalumab, tremelimumab, and chemotherapy interventional arm. The results from this cohort are pending. However, press releases indicate that this arm did show a statistically significant improvement in OS (66).

### Pembrolizumab

In Keynote-028, investigators assessed the safety and preliminary efficacy of pembrolizumab 10 mg/kg every 3 weeks in patient with extensive stage small cell lung cancer progressing on available standard of care therapies (67). Patients had tumors with PD-L1 expression ≥ 1% as measured by the 22C3 antibody. Primary endpoints included safety, tolerability, and ORR. The study was powered to detect with 80% power an ORR exceeding 10%. Efficacy analysis was conducted on all patients who received at least one dose of pembrolizumab. Twenty-four patients enrolled on the study and were eligible for the final efficacy analysis. Of these, one patient had a complete response, and seven patients enjoyed partial responses. The ORR was 33% (95% CI, 16–55%). Based on this data, the FDA approved pembrolizumab for use in patients with ES-SCLC which progressed on platinum-based chemotherapy and at least one other line of therapy.

A subsequent Phase 2 basket study, Keynote-158 assessed the efficacy of pembrolizumab in several tumor types, including advanced or extensive stage small cell lung cancer (68). As opposed to KN-028, patients could enroll irrespective of PD-L1 expression levels. Similar to KN-028, these patients all had tumors progressing on available standard therapies. One hundred and seven patients enrolled, with 47% patients expressing no PD-L1 in tumor samples. Only 39% had PD-L1 positive tumors, with the remainder of patients with unknown PD-L1 status. The pooled ORR was 18.7% (95% CI, 11.8–27.4%). Looking at PD-L1 subgroups, the response rate was higher, 35.7% (95% CI, 21.6–52%) in PD-L1 expressing patients while those with negative PD-L1 expression demonstrated an ORR of 6%. The median PFS in this population of patients was 2.0 months (95% CI, 1.9–2.1 months). However, the duration of response had not been reached at the time of this report, and 12 patients enjoyed a DOR ≥ 9 months. Pooled analysis of the KN-028 and 158 trials did not yield appreciably different results (69). However, this provided updates of several efficacy endpoints. The median DOR has still not been reached, and nine patients demonstrated DOR ≥ 18 months. Twelve and 24-months OS rates were 34 and 21%, respectively.

Other studies have studied additional approaches utilizing pembrolizumab, including as maintenance therapy. In a Phase 2 study, investigators assessed maintenance pembrolizumab in patients with extensive stage small cell lung cancer who demonstrated at least stable disease following completion of induction platinum and etoposide (70). Patients could enroll following PCI or consolidative thoracic radiotherapy. Patients received pembrolizumab 300 mg IV every 3 weeks, which was initiated within 8 weeks following completion of chemotherapy. Patients could enroll regardless of PD-L1 expression levels in the tumor or stroma. Overall, 45 patients enrolled with only 3 expressing PD-L1 on tumor cells and 8 expressing PD-L1 in the stroma. The median PFS was 1.4 months (95% CI, 1.3–2.8 months). This outcome did not appear to be an improvement beyond historical data.

Investigators have also assessed pembrolizumab in combination with platinum and etoposide for first-line treatment of patients with extensive stage small cell lung cancer (NCT03066778, Keynote-604). Patients could receive PCI, but not consolidative thoracic radiation, on this study following completion of the induction cycles of treatment. As of this writing, Keynote-604 met its first endpoint of median PFS but did not improve OS when compared to patients receiving chemotherapy alone (Merck 1/6/2020).

### Summary of the Evidence and Limitations

The IMpower133 and CASPIAN studies represent a new standard of care in the first-line management of ES-SCLC. The outcomes of the two trials are widely similar. However, they differed in several areas of trial design. Unlike the IMpower 133 regimen, CASPIAN allowed up to 6 cycles of D+CT in patients receiving carboplatin. IMpower 133 did not allow investigators a choice between cisplatin and carboplatin, either. Additionally, CASPIAN was open-label as opposed to a placebo-controlled trial. Finally, the CASPIAN trial contained a third arm which contained tremelimumab. This trial assigned patients randomly in a 1:1:1 fashion to D+CT, D+T+CT or CT alone, and the results of the D+T+CT arm were not reported in the paper by Paz-Aras, et al. (4) Prior experience, as discussed previously, generally demonstrated that the combination of D+T was safe and tolerable.

The role of combination CTLA-4 and PD-1/PD-L1 in the management of ES-SCLC is unclear when judging the totality of the evidence. Though the final results from the CASPIAN study in the durvalumab and tremelimumab arm are still pending, the current evidence on combination ipilimumab and nivolumab is instructive. Though response rates with combination ipilimumab and nivolumab are higher than can be expected with nivolumab monotherapy, this difference did not translate to OS benefit. The tolerability of combination ipilimumab and nivolumab may be an issue, with nearly one-third of patients experiencing grade 3 and 4 toxicities or discontinuing therapy due to treatment related adverse events.

The timing of initiation of checkpoint inhibitor therapy varied between several studies discussed. Studies of phased administration of checkpoint inhibitors or maintenance-only therapy, following completion of standard chemotherapy, also offer an interesting perspective on the benefit of checkpoint inhibitor therapy. Both Checkmate-451 and Gadgeel et al. (57, 70) did not show survival benefits using these strategies in patients with ES-SCLC. Investigators used the phased approach in Checkmate-551 based upon favorable results of this approach compared to initiation of ipilimumab with induction in a trial with carboplatin and paclitaxel. This result suggested a benefit to initial cytoreduction, enhancing subsequent immune activity, and it may have helped inform the design of subsequent maintenance-only studies (54). The positive findings from IMpower133 and CASPIAN suggest early administration of anti-PD-L1 therapy is optimal. Keynote-604, however, did not reproduce this benefit with anti-PD-1 therapy. Understanding of this discrepancy is pending full results from this trial. Given the current data, initiation of checkpoint inhibitors with induction platinum and etoposide is the most evidenced approach.

## Current Areas of Investigation

### Consolidative Radiotherapy

Consolidative thoracic radiotherapy has employed following completion of induction chemotherapy based upon several studies (71). These studies have shown some survival advantages to thoracic radiation in selected patients, particularly those with complete or excellent responses to chemotherapy who also have residual thoracic disease and minimal to no extra-thoracic sites of disease (72, 73). However, patients were not allowed to have consolidative radiation in the studies investigating the combination of checkpoint inhibitors with platinum-based chemotherapy in the first-line treatment of ES-SCLC. Whether this approach is correct is unclear. Data from the treatment of Stage III, unresectable NSCLC suggests that consolidation durvalumab following completion of concurrent chemoradiotherapy is safe, and this data clearly demonstrated survival advantages. However, it is unclear whether thoracic radiation following combination chemoimmunotherapy could lead to higher rates of pneumonitis, post-radiotherapy empyema, etc. Our institutional practice has been to select patients carefully for discussions regarding and treatment with consolidative radiotherapy.

Five active or planned studies are currently investigating this question (74). In NCT02934503, investigators are adding pembrolizumab to standard of care chemotherapy and consolidative radiotherapy. Pembrolizumab can begin, depending on the arm, with Cycle 1 or 2 of standard chemotherapy, following completion of induction chemotherapy or following completion of chemotherapy and consolidative radiotherapy. In NCT02402920, investigators are enrolling patients with both LS-SCLC and ES-SCLC, and those with ES-disease will get pembrolizumab following chemotherapy and starting with consolidative radiotherapy. Both NCT03923270 and NCT03043599 are exploring combination CTLA-4 and PD-1/PD-L1 inhibition with radiation. In NCT03043599, ipilimumab 3 mg/kg is initiated concurrently with consolidative radiotherapy and given every 3 weeks for four doses. This is then followed by nivolumab maintenance. NCT03923270 is utilizing either durvalumab monotherapy, tremelimumab with durvalumab, or durvalumab with olaparib after the completion of consolidative thoracic radiation.

### Limited Stage Disease

The treatment paradigm for the majority of cases of limited stage small cell lung cancer (LS-SCLC) involves concurrent chemoradiotherapy with platinum, etoposide chemotherapy (75). There are currently four studies either planned, or active which are investigating the role of checkpoint inhibitors in limited stage disease (74). Primarily, two approaches are seen. First, checkpoint inhibitors can complement standard chemoradiotherapy as sequential consolidation therapy. Second, investigators can incorporate checkpoint inhibitors in the standard concurrent regimen. NCT04189094 is investigating induction sintilimab (PD-1 inhibitor) with standard of care cisplatin, etoposide for 2 cycles (76). Concurrent chemoradiotherapy will utilize cisplatin and etoposide alone, followed by maintenance sintilimab. The STIMULI trial (NCT02046733), similarly to Checkmate-451, is assessing the efficacy of N1I3 when given after standard concurrent chemoradiotherapy. ADRIATIC (NCT03703297) mirrors STIMULI with durvalumab and tremelimumab. As opposed to induction or maintenance strategies alone, NCT03811002 is comparing chemoradiotherapy with or without the addition of atezolizumab throughout the entire treatment phase—including concurrent with radiation. In NCT02402920, investigators are enrolling patients with both LS-SCLC and ES-SCLC, and those with LS-disease will receive pembrolizumab beginning with concurrent radiotherapy. The results of these two studies, specifically, may help inform decision regarding the safety of consolidative radiotherapy in ES-SCLC in the consolidation setting.

### Other Combination Approaches With Checkpoint Inhibitors

Immune checkpoint inhibitors are firmly established in the treatment of recurrent ES-SCLC, and they have a newly established foothold in the treatment of newly diagnosed ES-SCLC. Current trials are investigating combination regimens with currently approved checkpoint inhibitors, namely with inhibitors of alternative immune checkpoints, small molecule inhibitors, additional immunomodulatory pathways, and cytotoxic chemotherapy agents. We briefly highlight examples of these approaches, and Table 1 presents a more detailed review of ongoing studies.

**Table 1:** Summary of Phase 3 trials investigating checkpoint inhibitors in ES-SCLC^a^.

**Trial**	**IMpower133**	**CASPIAN**	**Checkmate 451**		**CA184-156**	**Checkmate 331**
**(References)**	**(60)**	**(4)**	**(****57****)**		**(54)**	**(52)**
N	403	537	834		1,132	569
Population	1st-line, ES-SCLC	1st-line, ES-SCLC	1st-line, ES-SCLC		1st-line, ES-SCLC	2nd-line ES-SCLC
Checkpoint inhibitor(s) used	Atezolizumab	Tremelimumab Durvalumab	Ipilimumab Nivolumab	Nivolumab	Ipilimumab	Nivolumab
HR for OS (95% CI)	0.70 (0.54–0.91)	0.73 (0.59–0.91)	0.92 (0.75–1.12)	0.84 (069−1.02)	0.94 (0.81–1.09)	0.86 (0.73–1.04)
HR for PFS (95% CI)	0.77 (0.62–0.96)	0.78 (0.65–0.94)	0.72 (0.60–0.87)	0.67 (0.56–0.81)	0.85 (0.75–0.97)	1.41 (1.18–1.69)
% ORR intervention/control	60.2/64.4	68/58	NR	NR	62/62	39/47
% Gr ≥ 3 AE All Cause intervention/control	67.2/65.8	62/62	NR^b^	NR^b^	48/44	4/73^c^
# Treatment related deaths intervention/control	3/3	1/0	7/1	1/1	5/2	2/3

a *Keynote 604 full data not reported*.

b *All grades reported as 86% in ipilimumab, nivolumab arm vs. 61% in nivolumab vs. 50% in placebo*.

c *Treatment related*.

The response rates in an unselected population of patients with ES-SCLC appear to be modest, and combination approaches seek to augment these modest response rates. One approach includes combinations with antibodies targeting the immune checkpoints LAG-3 and TIM-3 (77). Ongoing trials are investigating combination PD-1 and LAG-3 inhibitors; bispecific inhibitors of PD-1/TIM-3 and CTLA-4/LAG-3; and single agent TIM-3 inhibitors (77, 78). T-cell immunoglobulin and ITIM domain (TIGIT) is also an emerging checkpoint for anti-cancer therapy. Activated T-cells and NK cells express TIGIT, and CD155 acts as a ligand (79). CD155, expressed on tumor stromal cells, can interact with TIGIT and induce immunosuppression. Blockade of TIGIT can induce CD226 co-stimulation of T-cells and NK cells.

Beyond checkpoint inhibitors, other immunomodulatory agents can affect innate immunity and therefore induce anti-tumor activity. BNT-411, for example, is a TLR7 agonist which can stimulate innate immune responses (80). ALT-803 is a fused complex of IL-15/IL-15Rα and IgG1. IL-15 has a similar mechanism of action at IL-2, and they signal through shared receptors. ALT-803 has been studied in NSCLC, and currently is under investigation in SCLC (81).

Small molecular inhibitors offer another strategy, and several clinical trials are investigating such combinations. One such molecule is trilaciclib, a CDK4/6 inhibitor (82). Trilaciclib, when used with chemotherapy, may improve chemotherapy response rates and protect hematopoietic precursors and other immune cells. NCT03041311 is investing combination carboplatin, etoposide, atezolizumab, and trilaciclib in ES-SCLC. PARP inhibitors have demonstrated, though modest, activity in SCLC patients (83). In addition to combinations with other cytotoxic agents, olaparib is being studied in combination with durvalumab. Finally, BXCL-701 is a DPP8/9 and FAP inhibitor has been shown to upregulate pro-inflammatory cytokines and immune effector cells in colon cancer models when given in combination with CTLA-4 and TGFβ inhibitors (84). Though it is a radiolabeled (^177^Lutetium) somatostatin analog and not a small molecule, 177Lu-DOTA0-Tyr3-Octreotate (Lutathera) offers the ability to target somatostatin receptors expressed on some neuroendocrine tumors, including SCLC. This agent is being studied in combination with nivolumab (85).

Cytotoxic chemotherapy remains an essential component of treatment for ES-SCLC, and immune checkpoint inhibitors may synergize with agents other than platinum and etoposide. Lurbinectedin is one such agent that induces degradation of RNA polymerase II, inducing the accumulation of DNA breaks (86). It has demonstrated promising activity for treatment of recurrent small cell lung cancer, with a pooled ORR of 35.2% (95% CI, 26.2–45.2) (87). Patients with platinum resistant disease demonstrated response rates of 21.3% (95% CI, 10.7–35.7), and those with platinum sensitive disease had an ORR of 46.6% (95% CI, 33–60.1). This agent is being tested in combination with atezolizumab. Pegzilarginase is a human arginine degrading enzyme with anti-tumor effects, potentially due to starving arginine “addicted” tumor cells. It may also increase CD8+ T-cell recruitment into tumors (88). Clinical trials are also combining additional cytotoxic chemotherapy agents with checkpoint inhibitors, including amrubicin, temozolomide, and irinotecan highlighting future directions of immunotherapy in SCLC.

## Future Directions of Immunotherapy in SCLC

### Cellular Therapy

Adoptive cellular therapy (ACT) involves the infusion of T-cells into patients with the aim of kindling a tumor-specific immune response and has shown promise in various cancers by its ability to provide an immune infiltrate to be trafficked into “poorly inflamed” tumors. A variation on ACT known as chimeric-antigen receptor T cell (CAR-T) therapy involves engineered T-cells with tumor-antigen specificity and has recently been widely employed with significant success in hematologic malignancies, including acute lymphoblastic leukemia (89). A phase 1 clinical trial using CAR-T cells with specificity for DLL-3 (AMG 119) is currently underway (NCT03392064). A similar strategy targeting DLL3, using the bispecific T-cell engager (BiTE) AMG 757 (NCT03319940), is also underway. A variety of clinical trials using CAR-T therapies are being developed recently in various solid tumors, with the expectation that this approach will be investigated further in SCLC.

### Next Generation of Vaccines and Combinations With Immune Checkpoint Inhibitors

Although clinical success with anti-tumor vaccinations in SCLC had historically been limited, the signals of antitumor activity in certain subgroups of patients, coupled with new excitement surrounding checkpoint blockade as a means to augment the response to vaccination, have revived significant interest in this area of immunotherapy. More recently, the combination of the Ad.p53 DC vaccine with nivolumab and ipilimumab is currently under investigation for relapsed SCLC (NCT03406715). Another trial is investigating the use of galinpepimut-S vaccine (a WT1 analog peptide vaccine) in combination with pembrolizumab in multiple tumor types including SCLC (NCT 3761914). More of these combination trials will likely be developed in the coming years.

### Biomarker Development

Given the relatively low response rates seen with currently available checkpoint inhibitors, predictive biomarkers of response to immunotherapy are of significant interest to guide treatment selection. At present, PD-L1 expression and tumor mutational burden (TMB) are the best-characterized and commonly used biomarkers for immunotherapy response in SCLC.

Tumor-cell positivity for PD-L1 has been shown to be significantly less frequent in SCLC as compared to non-small cell lung cancer (50, 67). In the CheckMate-032 trial, PD-L1 expression was assessed using the tumor proportion score (TPS), or the percentage of tumor cells with positive staining by immunohistochemistry. Response rates to either nivolumab monotherapy or in combination with ipilimumab did not differ significantly between patients who had PD-L1 positive vs. PD-L1 negative tumors (although not all patients were evaluable for PD-L1 expression) (50). In contrast to this finding, as part of a combined analysis of KEYNOTE-028 and KEYNOTE-158 trials, PD-L1 positivity did correlate with clinical benefit from pembrolizumab, with 14 out of 16 responders being PD-L1 positive (69). In the KEYNOTE trials, PD-L1 expression was assessed as combined positive score (CPS), or the total number of tumor cells plus associated immune cells staining positive for PD-L1 divided by the number of viable tumor cells. The difference in technique for measuring PD-L1 expression could potentially explain these conflicting observations. Whether the CPS may be a better predictor than TPS for response to immunotherapy in SCLC remains unclear and may require further investigation.

A correlative analysis of TMB and outcomes in the CheckMate-032 trial demonstrated enhanced efficacy of nivolumab with or without ipilimumab in patients with high TMB as compared to those with low TMB. In addition, patients within the highest tertile of TMB appeared to have the greatest benefit from the combination (46.2% response rate) vs. nivolumab monotherapy (21.3% response rate) (90). Of note, in the IMpower-133 trial, a blood-based TMB measurement did not correlate with outcomes with the addition of atezolizumab (91). Further study is clearly needed to identify the optimal way to measure these and other biomarkers to predict response to various immunotherapies.

## Conclusion

The development of immunotherapies as treatment for SCLC has been many years in the making, although early results using cytokine and vaccine treatments were met with limited success. Only recently has the discovery and incorporation of checkpoint inhibitors led to breakthroughs in this area, representing the biggest advances in SCLC treatment in decades. These discoveries have established a new standard of care and spurred a revival in immunotherapy research for this disease. Current and future efforts are underway to explore combinations of immunotherapies, small-molecule, and chemotherapy combinations with immunotherapy, as well as biomarkers for selection of immunotherapies.

## Author Contributions

AS and MS contributed equally as first authors to this chapter. Specific contributions include the pre-immune checkpoint to checkpoint timeline, cytokine and vaccine therapies, combination vaccination plus checkpoint, and biomarkers for immune-oncologic response. AC is the senior and corresponding author contributing equally to the oversight, draft, and review of the chapter. All authors contributed to the article and approved the submitted version.

## Conflict of Interest

MS serves as the site Principal Investigator for multiple trials receiving research support from the following sponsors: Merck/Serono, Vaccinex, Nektar Therapeutics, PsiOxus Therapeutics, Amphivena, and Genentech. MS has also received travel support from Janssen Research and Development. AC has accepted consulting/advisory roles with Novartis, AstraZeneca, and Pfizer, and speaking engagements with Genentech, Takeda, Celgene, and Novartis. AC's research has also been sponsored by Novartis, Bristol-Myers Squibb, and AstraZeneca. AS has received travel sponsorship from Daiichi Sankyo and Prime Oncology. AS also maintains an advisory role with AstraZeneca. The handling editor declared a past relationship with the authors.
